# Research Progress on Marine Active Substances in Improving Atherosclerosis

**DOI:** 10.3390/md24050165

**Published:** 2026-05-06

**Authors:** Chenyang Xie, Jiapeng Qi, Wenmei Huang, Bei Chen, Min Xu, Shuji Liu, Yongchang Su, Yixiong Lin, Kun Qiao, Zhiyu Liu

**Affiliations:** 1 Engineering Technological Center of Mushroom Industry, School of Biological Science and Biotechnology, Minnan Normal University, Zhangzhou 363000, China; m18652928716@163.com; 2 Key Laboratory of Cultivation and High-Value Utilization of Marine Organisms in Fujian Province, Fisheries Research Institute of Fujian, Xiamen 361013, China; 15535285741@163.com (J.Q.); chenbeifjfri@foxmail.com (B.C.); xumin@jmu.edu.cn (M.X.); cute506636@163.com (S.L.); suyongchang@126.com (Y.S.); 3 Fisheries College, Jimei University, Xiamen 361021, China; 4 Xiamen Daozhiyuan Biological Technology Co., Ltd., Xiamen 361024, China; daozhiyuan188@163.com

**Keywords:** marine-derived active substances, atherosclerosis, molecular mechanism

## Abstract

Atherosclerosis is the primary pathological foundation of various cardiovascular and cerebrovascular diseases. Although existing treatment strategies exhibit certain efficacy, they still encounter limitations such as pronounced side effects and a single-target approach. The oceans have nurtured a rich diversity of organisms, and the secondary metabolites they generate possess novel structures, diverse activities, and unique mechanisms of action, offering new prospects for the development of anti-atherosclerotic drugs. This paper reviews advancements related to research on marine-derived active substances that possess anti-atherosclerotic activity, as well as current challenges in research on active substances, with the objective of laying a foundation for the development of anti-atherosclerotic drugs.

## 1. Introduction

Cardiovascular diseases (CVD) have progressively emerged as one of the primary threats to global life and health, accounting for approximately 32% of all global mortalities in 2022 [1]. Among these, atherosclerosis serves as a crucial pathological foundation that triggers a multitude of fatal diseases [2]. Atherosclerosis is a chronic ailment of the arterial vessel wall and is characterized by the aberrant accumulation of lipids, inflammatory cells, smooth muscle cells, and extracellular matrix beneath the arterial intima [3].

**Figure 1:**
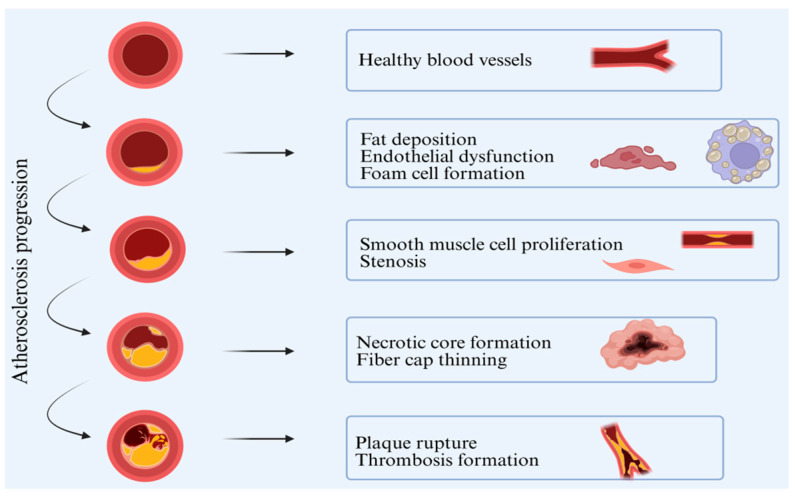
Process of development of atherosclerosis. Four stages of vascular changes during atherosclerosis: early endothelial dysfunction and foam cell formation, middle-stage proliferation of smooth muscle cells and vascular stenosis, late-stage necrotic fibrous plaque formation, ultimately leading to thrombus formation.

The onset of atherosclerosis is a complex process commencing with excessive accumulation of lipids, which results in dysfunction of endothelial cells [4]. As shown in Figure 1, this process recruits monocytes and induces their differentiation into macrophages. Subsequently, macrophages engulf lipids to form foam cells; this process is accompanied by the onset of chronic inflammatory responses [5]. Simultaneously, smooth muscle cells undergo phenotypic transformation, proliferation, and migration, and form necrotic plaques along with macrophages and other components. These plaques are highly susceptible to being ruptured, thereby leading to thrombosis [6].

**Figure 2:**
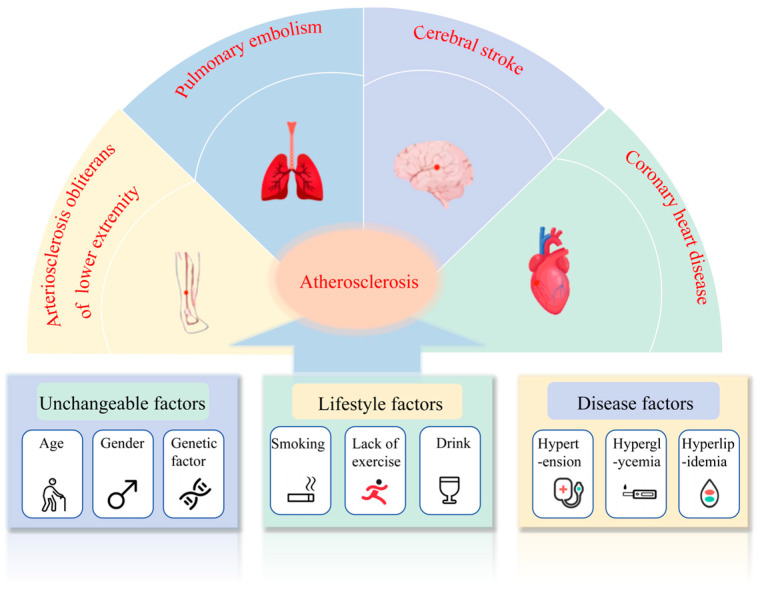
Causes of Atherosclerosis and Related Diseases. Factors such as being male, elderly age, family history, smoking, drinking, lack of exercise, hypertension, hyperglycemia, and hyperlipidemia are prone to cause this disease. Atherosclerosis will eventually develop into stroke, myocardial infarction, pulmonary embolism, and atherosclerotic occlusive disease of the lower extremities.

As shown in Figure 2, atherosclerosis is primarily induced by factors, such as hypertension, hyperlipidemia, hyperglycemia, smoking, obesity, and aging [7,8]. Currently, drugs employed for the treatment of atherosclerosis predominantly target lipid-lowering, anti-inflammatory, and anticoagulant effects, as exemplified by the use of atorvastatin and aspirin. Nevertheless, these drugs commonly exhibit adverse reactions, such as hepatotoxicity, gastrointestinal discomfort, and myalgia [9,10]. Moreover, individual drugs are inadequate for tackling the series of symptoms that stem from atherosclerosis. Consequently, to prevent or ameliorate atherosclerosis, there is an urgent need to develop natural drugs that exhibit minimal side effects and possess multi-target properties.

Oceans encompass approximately 70% of the surface area of the Earth. In contrast to terrestrial ecosystems, the oceans feature a distinctive ecosystem characterized by low temperatures, low oxygen levels, low light intensity, high pressure, and high salinity, which sustain approximately 10,000 known marine species. Under such harsh environmental conditions, these organisms synthesize or accumulate chemical substances with notable biological activities within their physiological systems [11]. These substances offer a highly promising reservoir of raw materials for synthesis of drugs that can be used in humans [12].

Currently, through extraction, separation, and identification processes of active substances, numerous marine-derived bioactive substances have already been demonstrated to exhibit anti-inflammatory [13], antioxidant [14], immunoregulatory [15], and antiviral [16] activities, among others. Consequently, an increasing number of researchers are employing such substances to develop drugs that target atherosclerosis. This paper reviews marine-derived active substances relevant to atherosclerosis, elaborates on their structure activity relationships and mechanisms of action, and provides insights into the development of novel drugs aimed at ameliorating atherosclerosis.

## 2. Marine-Derived Active Substances in Anti-Atherosclerosis

**Figure 3:**
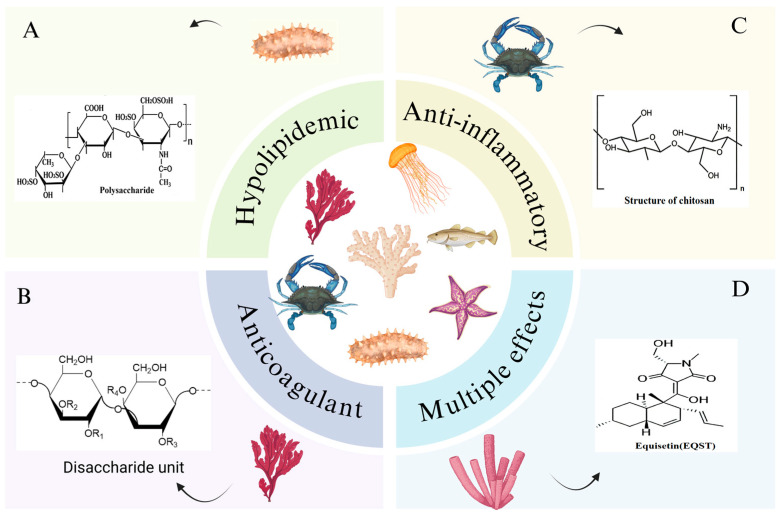
Marine-derived bioactive substances and their structures. (**A**) Structure of sea cucumber polysaccharides extracted from sea cucumbers. (**B**) Structure of polysaccharides extracted from Seaweed. (**C**) Structure of chitosan extracted from blue crab shells. (**D**) Structure of equisetin extracted from sponge fungi.

Owing to the harsh marine environment as well as competitive relationships and defense requirements among organisms, marine organisms have been compelled to evolve distinct biosynthetic pathways compared to those of terrestrial organisms. Consequently, marine organisms have developed biosynthetic pathways that differ from those of terrestrial organisms, resulting in a wide variety of metabolic products, such as special functional groups, complex macrocyclic esters, and unique amino acid compositions. As shown in Figure 3, these structures endow marine organisms with strong biological activities, such as lipid-lowering, antihypertensive, anti-inflammatory, antioxidant, and anticoagulant effects [17,18,19,20], which render them as potential raw materials for the treatment of atherosclerosis.

### 2.1. Lipid-Regulating Active Substances

Atherosclerosis occurs when elevated levels of low-density lipoprotein cholesterol infiltrate blood vessels, accumulate in the intima of arteries, and initiate a succession of pathological processes [21]. Research has demonstrated that numerous bioactive substances of marine origin possess lipid-lowering effects. For example, Wu et al. extracted polysaccharide (SFPS) and ultrasonically degraded polysaccharide (SFPSUD) from *Sargassum fusiforme*. In zebrafish fed a high-fat diet, multiple concentrations were tested (larvae: 200 mg/L, 600 mg/L; adults: 20 mg/L, 50 mg/L). The measured parameters included total cholesterol (TC), triglycerides (TG), hepatic lipase (HL), lipoprotein lipase (LPL), and the change rate of condition factor. The results showed that SFPS reduced TC/TG and increased HL and LPL activities, with SFPSUD exhibiting a greater improvement. Specifically, at 50 mg/L, the SFPS group showed a 25.53% reduction in condition factor, a 153.00% increase in HL, and a 123.48% increase in total lipase (TL); in the SFPSUD group at the same concentration, the corresponding values were a 27.18% reduction in condition factor and 168.20% and 137.73% increases in HL and TL, respectively [22].

Sulfated polysaccharides obtained from *Pearsonothuria graeffei* and *Isostichopus badionotus* through enzymatic hydrolysis significantly inhibited pancreatic lipase activity. By analyzing mouse serum, these polysaccharides were found to effectively elevate the content of high-density lipoprotein (HDL) and reduce the content of low-density lipoprotein (LDL). Several sulfated polysaccharides extracted from sea cucumbers maintain an irregularly coiled chain conformation in aqueous solution. This chain conformation endows these polysaccharides with distinct physicochemical properties and enables them to exhibit high apparent viscosity. Different chain conformations result in different lipid-lowering activities [23].

In addition to certain polysaccharides discovered in marine organisms that live in the ocean, some unsaturated fatty acids also possess the ability to reduce blood lipid levels. Phospholipids are abundant in docosahexaenoic acid (DHA) and eicosapentaenoic acid (EPA), both of which are known to effectively lower blood lipid levels and safeguard the cardiovascular system. Ding et al. extracted EPA-enriched ethanolamine plasmalogen (EPA-PlsEtn) from marine sources. This compound features an O-cis-alkenyl ether bond at the sn-1 position and EPA at the sn-2 position. In LDLR^−/−^ mice, treatment with EPA-PlsEtn for 8 weeks reduced atherosclerotic lesion area by 78%, serum LDL-C by 73.9%, total cholesterol by 33.6%, and LDL-C by 38.2%, while faecal total neutral sterols and bile acids increased by 92% and 39%, respectively. Mechanistically, EPA-PlsEtn promoted cholesterol conversion to bile acids via upregulation of CYP7A1. Compared with ethyl ester EPA (EPA-EE), EPA-EE did not exhibit similar cholesterol-lowering effects, suggesting that the unique plasmalogen structure plays a critical role in these activities [24]. Moreover, in a randomized controlled trial involving patients with obesity-related dyslipidemia, Saraswathi et al. evaluated the lipid-lowering and anti-inflammatory effects of a 12-week combination regimen of omega-3 polyunsaturated fatty acids (2 g twice daily) and the COX inhibitor naproxen (220 mg twice daily). Serum triglyceride levels showed a decreasing trend in the omega-3 monotherapy group, whereas a significant reduction was achieved in the omega-3 plus naproxen combination group (*p* < 0.05) [25].

Active peptides are referred to as small peptide segments with specific regulatory functions. They are extracted and isolated by employing techniques such as enzymatic hydrolysis and microbial fermentation. Owing to their low molecular weight and ease of digestion and absorption by the human body, marine-sourced active peptides are gradually being exploited by humans [26]. They exhibit abundant biological activities, and lipid-lowering activity is one of them. For example, a collagen peptide obtained through enzymatic hydrolysis from mackerel contained a high proportion of arginine, aromatic, and hydrophobic amino acids, and had a well-defined and uniform fiber structure, endowing it with good biological activity. In a high-fat zebrafish model, collagen peptides effectively reduced the weight and body width of zebrafish. Their lipid-lowering abilities were further verified by measuring changes in four blood lipid parameters [27].

### 2.2. Antioxidant Stress-Resisting and Anti-Inflammatory Active Substances

During development of atherosclerosis, a series of inflammatory reactions occur due to accumulation of lipids. Within cells, elevation of cholesterol and triglycerides results in impairment of various intracellular organelles, leading to generation of a substantial quantity of reactive oxygen species. Therefore, anti-inflammatory and antioxidant substances can effectively impede the progression of atherosclerosis [28].

Ye et al. isolated a sulfated polysaccharide, SCVP-2 (molecular weight 209.1 kDa, sulfate content 25.5%), from the viscera of the sea cucumber *Apostichopus japonicus*. In LPS-induced RAW264.7 macrophages, SCVP-2 at 50 and 100 μg/mL reduced NO production to 61.8% and 37.5% of the model group, respectively; at 200 μg/mL, the NO inhibition rate reached 85.9% (IC_50_ = 61.3 μg/mL). In a xylene-induced mouse ear edema model, intragastric administration of SCVP-2 (50 and 100 mg/kg) resulted in edema inhibition rates of 45.6% and 68.3%, respectively, comparable to the positive control indomethacin (10 mg/kg, inhibition rate 72.1%). These results demonstrate that SCVP-2 possesses significant anti-inflammatory activity both in vitro and in vivo [29]. Polysaccharides extracted from oysters [30] contain a large amount of glucose and can effectively inhibit colitis induced by dextran sulfate sodium (DSS), augment the expression of anti-inflammatory factors in the intestine, and maintain stability of the intestinal microbiota and intestinal barrier in mice.

Active oxygen encompasses substances such as nitric oxide, hydrogen peroxide free radicals, hydroxyl free radicals, and oxygen anions. Oxidative damage significantly influences the progression of atherosclerosis. A report suggests that chitosan extracted from *Portunus trituberculatus* [31] possesses amino and carboxyl functional groups, which are commonly recognized as primary active groups with antioxidant potential. The 2,2′-Azinobis-(3-ethylbenzothiazoline-6-sulfonic acid) method confirmed that chitosan can scavenge free radicals and exhibit antioxidant activity. Lutein and fucoxanthin extracted from algae [32] are abundant in *Z*-isomers, which confer antioxidant properties upon them. Experimental findings indicate that these two antioxidants can effectively eliminate singlet oxygen, superoxide anions, hydroxyl radicals, and lipid peroxidation. Yellowfin tuna [33] is rich in selenium. Selenium-rich peptides can be obtained via enzymatic hydrolysis. These peptides can reduce the levels of malondialdehyde and reactive oxygen species in cells, effectively mitigating the oxidative stress response in cells.

### 2.3. Substances That Possess Anticoagulant Activity

In the advanced stages of atherosclerosis progression, apoptosis of diverse cell types gives rise to a necrotic core, which induces platelet aggregation and blood coagulation, ultimately leading to formation of a thrombus [34]. Consequently, numerous researchers have focused on substances that possess anticoagulation activity for the treatment of atherosclerosis. Furthermore, many substances identified in marine-derived active substances have also been found to possess anticoagulant characteristics.

For example, Ustyuzhanina et al. [35] isolated an oversulfated dermatan sulfate fraction (LA-F1), a heparinoid fraction (LA-F2), and its reduced derivative (LA-F1-RED) from the body wall of *Lysastrosoma anthosticta*. These fractions contain abundant 2,3-di-*O*-sulfated iduronic acid residues. In the evaluation of in vitro anticoagulant activity, the activated partial thromboplastin time (APTT) assay showed that the concentrations required to double the APTT (2× APTT) were 2.00 ± 0.10 μg/mL for LA-F1, 1.52 ± 0.11 μg/mL for LA-F2, and 3.94 ± 0.13 μg/mL for LA-F1-RED, whereas the positive control heparin gave a value of 0.85 ± 0.05 μg/mL. Among these, the activity of LA-F2 was the closest to that of heparin and was higher than those of LA-F1 and LA-F1-RED. Furthermore, LA-F1 and LA-F1-RED still exhibited significant anti-IIa and anti-Xa activities, indicating that the IdoA2S,3S or IdoA2S3S structural units are crucial for the anticoagulant effect.

Dong et al. extracted and purified chondroitin sulfate (CCS) from the bones of Pacific cod (*Gadus macrocephalus*). Structural characterization showed that CCS had good homogeneity, a low molecular weight (12.3 kDa), and was mainly composed of sulfated disaccharides. In terms of activity evaluation, this polysaccharide significantly prolonged both activated partial thromboplastin time (APTT) and thrombin time (TT) at concentrations of 5 μg/mL and 25 μg/mL, with prolongation multiples of 1.08-fold and 1.12-fold, respectively, confirming its clear in vitro anticoagulant activity at low concentrations [36].

Syed et al. [37] isolated and purified an anticoagulant peptide, VITPOR AI, consisting of 16 amino acid residues from *Porphyra yezoensis* and evaluated its inhibitory effect on coagulation factor XIIa (FXIIa) through in vitro experiments and molecular simulation analyses. A chromogenic substrate assay revealed that the peptide exhibited a half-maximal inhibitory concentration (IC_50_) of 70.24 μM against the amidolytic activity of FXIIa and an inhibition rate of 59.55% at a concentration of 0.1 mM. Molecular docking and 100 ns molecular dynamics simulations demonstrated that VITPOR AI binds via hydrogen bonds to key residues (Pro96, Tyr99, Glu146, Gly193, and Ser195) within the catalytic domain of FXIIa and interacts with the oxyanion hole of the catalytic pocket, resulting in stable binding without dissociating from the binding region throughout the simulation. This further elucidated the molecular mechanism underlying its inhibitory activity against FXIIa. Collectively, VITPOR AI exhibits clear FXIIa-targeted inhibitory activity at both the molecular and functional levels, providing structural and pharmacodynamic data that support its development as a novel anticoagulant candidate that acts specifically on the intrinsic coagulation pathway without interfering with the extrinsic pathway.

### 2.4. Multi-Target Synergistic Active Substances

Atherosclerosis is a complex process encompassing multiple factors and multi-link mechanisms, such as lipid metabolism dysregulation, endothelial dysfunction, and chronic inflammatory responses [38]. Traditional single-target treatment approaches have failed to yield significant outcomes. Multi-target therapy refers to influencing two or more genes or proteins within an entire signaling pathway, thereby modulating the entire pathway and enhancing anti-atherosclerotic efficacy. Marine bioactive substances exhibit remarkable therapeutic potential because of their anti-inflammatory, antioxidant, anti-thrombotic, and lipid-lowering activities [39]. Numerous marine bioactive substances possess multi-target synergistic activities and can simultaneously intervene in multiple aspects related to the development of atherosclerosis.

Polysaccharides extracted from *Gelidium crinale* [40] were found to contain substantial amounts of carbohydrate and sulfate groups, and they had relatively low molecular weights. Spectral analysis indicated the presence of sugar alcohols, sulfate groups, pyranose rings, and other special structures. These polysaccharides were found to efficiently eliminate reactive oxygen species and decrease the expression of proinflammatory factors, thereby demonstrating outstanding antioxidant and anti-inflammatory effects.

In addition to the fact that marine organisms possess a certain degree of anti-atherosclerotic activity, extracts obtained from marine fungi and seawater were also found to display certain levels of anti-atherosclerotic properties. For example, Radhakrishnan et al. [41] extracted organic matter (C18-DOM) from deep Pacific seawater using C18 solid-phase extraction. In vitro, treatment with C18-DOM significantly inhibited platelet aggregation, P-selectin expression, and COX-1 activity, while upregulating the expression of heme oxygenase-1 (HO-1), an anti-atherosclerotic marker, in endothelial cells. In a balloon-injured rabbit common carotid artery model, the neointima-to-media area ratio in the C18-DOM oral administration group (0.08 ± 0.05) was significantly reduced compared with the control group (1.86 ± 0.50), and the inhibitory effect was stronger than that of the 1% deep seawater group (0.32 ± 0.14), both with statistically significant differences (*p* < 0.05). In contrast, the deep seawater group containing only inorganic salts showed no significant inhibitory effect, indicating that the anti-atherosclerotic effect is attributed to the organic components of C18-DOM. Zhou et al. isolated and purified an extract, MFS (Metabolite of *Fusarium solani* FG319), from the fermentation products of the marine fungus *Fusarium solani* FG319, and evaluated its inhibitory effect on HMG-CoA reductase via an in vitro HMG-CoA reductase inhibition assay. At a test concentration of 100 mg/L, this extract achieved a maximum inhibition rate similar to that of the positive control lovastatin, and the overall inhibitory effect of MFS on HMG-CoA reductase was stronger than that of lovastatin [42].

## 3. Drug Efficacy Evaluation Model

Currently, anti-atherosclerotic effects of marine-sourced natural products can be evaluated using both in vivo and in vitro methods. In in vitro experiments, cells such as RAW264.7, THP-1, and HUVEC [43] are predominantly employed [44]. Modeling approaches mainly involve induction of cell inflammation via lipopolysaccharides or generating foam cells through oxidized low-density lipoproteins (ox-LDL).

**Figure 4:**
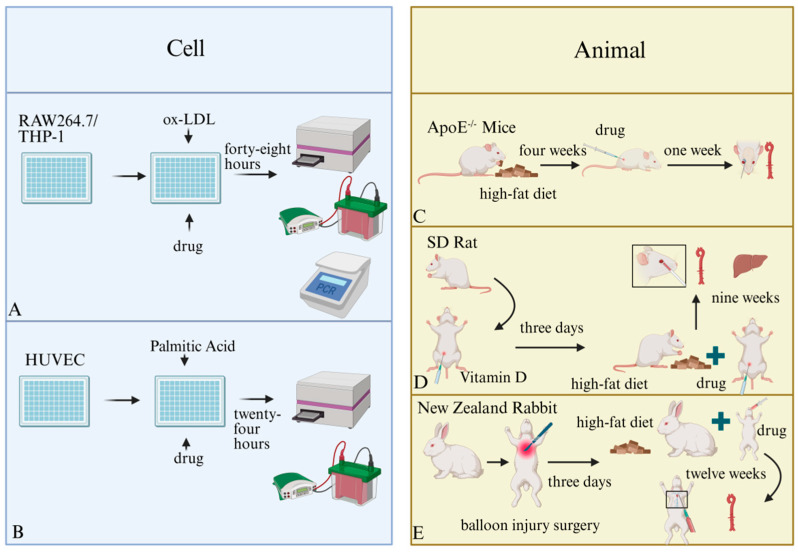
Model for Evaluation of Drug Efficacy. (**A**) Adding oxidized low-density lipoprotein to RAW264.7 or THP-1 cells can simulate the process of macrophage foam cell formation. (**B**) Adding palmitic acid to human umbilical vein endothelial cells (HUVECs) can simulate the process of endothelial cell dysfunction. (**C**) Atherosclerosis model in APOE^−/−^ mice induced by a high-fat diet. (**D**) Atherosclerosis model in rats induced by the combination of drugs and a high-fat diet. (**E**) Atherosclerosis model in New Zealand rabbits induced by the combination of surgery and a high-fat diet.

For in vivo verification, the animals selected mainly included ApoE^−/−^ mice, rats, New Zealand rabbits, and zebrafish. Success of the use of such models is mainly ascertained by measuring serum lipid concentrations and inflammatory factors [45]. In addition, methods such as measuring adhesion factors and oxidase activity have been utilized to evaluate efficacy of drugs [46]. Table 1 and Figure 4 present the active substances produced by certain marine organisms, along with the evaluation methods and results of their efficacy.

Bao et al. [44] corroborated the anti-atherosclerotic activity of sulfated dextran through the establishment of THP-1 cell and ApoE^−/−^ mouse models. After inducing differentiation of THP-1 cells into macrophages using phorbol myristate acetate (PMA), these cells were exposed to varying concentrations of the drug for 6 h, followed by addition of 50 μg/mL ox-LDL for 48 h. The findings demonstrated that the reactive oxygen species (ROS) content in the model group increased significantly. Polymerase chain reaction (PCR) and Western blotting (WB) indicated that sulfated dextran notably inhibited expression of the LOX-1 protein. In the ApoE^−/−^ mouse model, after the mice in the high-fat model group were fed a high-fat diet for four weeks, sulfated dextran was administered via subcutaneous injection for one week. The results revealed that serum lipid content of the mice in the drug-administrated group decreased significantly, and aortic plaques were significantly reduced.

He et al. assessed the protective efficacy of Dapagliflozin against endothelial injury by establishing a HUVEC model. Following incubation of HUVECs with palmitic acid (PA) and the drug for 24 h, effectiveness of the drug was gauged by measuring the expression of SIRT1 protein and conducting tube formation assays in the HUVECs. The findings indicated that the drug could modulate protein expression and reinstate the tube formation capacity of HUVECs [47].

Shi et al. established an atherosclerosis model in New Zealand rabbits. After a one-week adaptive feeding period, the rabbits underwent balloon injury surgery. Rabbits received oral administration of a combination of fucoidan and simvastatin while being fed a high-fat diet. After 12 weeks, blood samples were collected from the heart, and tissues including the aorta and liver were removed to perform experiments. The results demonstrated that blood lipid levels in rabbits in the treatment group were notably lower than those in the control group, and the area of aortic plaques was diminished [48].

Zhou et al. developed a Wistar rat model. After one week of adaptive feeding, the rats were injected with vitamin D3 for three days and subsequently fed a high-fat diet for 9 weeks. The injections were administered intraperitoneally daily. The results revealed that serum lipid content significantly decreased, expression of pro-inflammatory factors was reduced, and fat deposition stained with Oil Red O was less in the treated rats than in the control rats [46].

**Table 1:** Methods to Evaluate Therapeutic Effects of Marine-sourced Bioactive Substances.

Name	Source	Model	Outcome	References
Equisetin	*Fusarium equiseti*	BMDMs RAW264.7 ApoE^−/−^ mice	TC ↓, TG ↓ IL-6 ↓, TNF-α ↓	[49]
Astaxanthin	*Haematococcus pluvialis*	RAW264.7 ApoE^−/−^ mice	MDA ↓, GSH ↓ ROS ↓	[50]
Sulfated chondroitin A	*Laminaria japonica*	LDLr^−/−^ mice	TC, TG, LDL-C ↓, HDL-C ↑ TNF-α, IL-1β, IL-6 ↑, IL-10 ↑ SOD, MDA ↓	[51]
Alkaloid	*Aspergillus terreus*	HUVECs	IL-6, IL-1β, TNF-α ↓	[52]
Blue mussel hydrolysate	*Mytilus edulis*	RAW264.7	TNF-α, IL-6, TNF-α ↓ TC, FC, CE ↓, ROS ↓	[53]
Kelp polysaccharide	*Saccharina japonica*	THP-1 ApoE^−/−^ mice	SR-A1 ↓, ABCA1 ↑	[54]
Sea potato extract	*Acaudina molpadioides*	Hep-G2	PCSK9 ↓	[55]
Collagen Peptides	*Salmo salar*	RAW264.7 EA.hy926 ApoE^−/−^ mice	TC, TG, LDL-C ↓, HDL-C ↑ NO, IL-6, IL-1β, TNF-α ↓ MCP-1, SOD, MDA ↓	[56]
Kelp saponins	*Pearsonothuria graeffei*	Rat ApoE^−/−^ mice	TC, LDL-C ↓, HDL-C ↑ TNF-α ↓	[57]
Phospholipid	*Cucumaria frondosa*	ApoE^−/−^ mice	TC, TG, LDL-C ↓, HDL-C ↑ FC, CE ↓ IL-6, TNF-α↓	[24]
Kelp fermentation product	*Laminaria japonica*	HUVECs	IL-1β, IL-6, TNF-α ↓ MCP-1, VCAM-1, ICAM-1 ↓	[58]
Blue mussel peptide	*Mytilus edulis*	HUVECs	ROS ↓ caspase-3 ↓	[59]
Algal polysaccharides	*Fucus vesiculosus*	New Zealand rabbit	TC, TG ↓ LDLR, ABCG5 ↑	[48]

Note. Upward arrow (↑) denotes an increase in content, and downward arrow (↓) denotes a decrease.

## 4. Targeting Sites and Molecular Mechanisms

**Figure 5:**
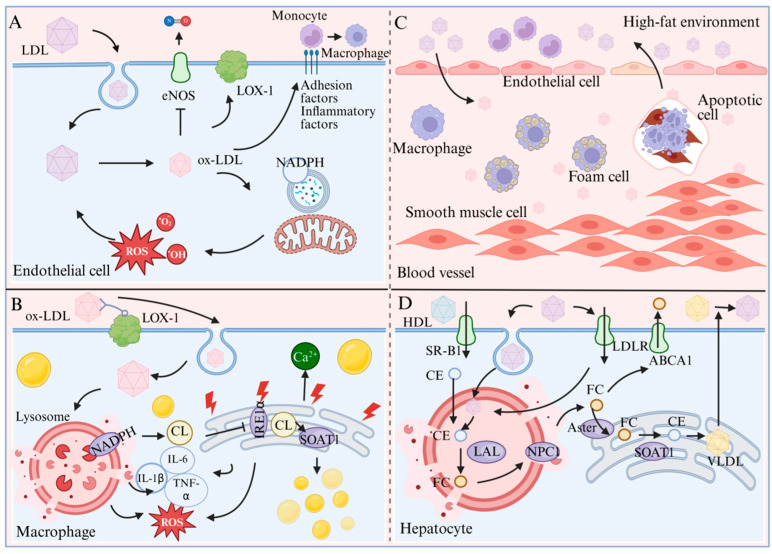
Molecular Mechanisms of Atherosclerosis (in endothelial cells, macrophages, blood vessels, and hepatocytes). (**A**) In a high-fat environment, endothelial cells undergo oxidative stress and gradually undergo apoptosis. (**B**) In a high-fat environment, macrophages foam and trigger inflammation, while endoplasmic reticulum stress produces reactive oxygen species. (**C**) Abnormal changes in endothelial cells, macrophages, and smooth muscle cells in a high-fat environment. (**D**) Hepatocytes produce large amounts of cholesterol in a high-fat environment.

Atherosclerosis is a multifactorial, multi-stage pathological process involving lipid metabolism disorders, endothelial dysfunction, chronic inflammation, and vascular remodeling, which is mediated by a series of key molecular targets and signaling pathways (Figure 5) [60]. Briefly, circulating LDL infiltrates the subendothelial space through a damaged endothelial barrier and is oxidized into ox-LDL. On the one hand, ox-LDL inhibits the activity of endothelial nitric oxide synthase (eNOS), impairing vascular relaxation function [61]; on the other hand, it induces reactive oxygen species (ROS) production via activating the LOX-1 receptor, exacerbating endothelial oxidative stress and inflammatory responses, thereby recruiting circulating monocytes to infiltrate and differentiate into macrophages [62]. Subsequently, macrophages take up ox-LDL in large quantities through LOX-1 receptors. After being degraded into free cholesterol in lysosomes, free cholesterol is esterified into lipid droplets via SOAT1-mediated cholesterol esterification in the endoplasmic reticulum, leading to progressive foam cell formation. This process is accompanied by NADPH oxidase-mediated ROS release and secretion of pro-inflammatory cytokines such as IL-1β, IL-6, and TNF-α, further amplifying the local inflammatory microenvironment [63]. Under persistent stimulation of a high-fat environment, apoptotic foam cells release abundant lipids and cellular debris, forming the necrotic core of plaques; meanwhile, vascular smooth muscle cells proliferate and migrate, contributing to the formation of the fibrous cap. Together, these events drive the initiation and progression of atherosclerotic plaques [64]. In addition, hepatocytes coordinately regulate cholesterol uptake, reverse transport, and esterification through key receptors and enzymes including SR-B1, LDLR, ABCA1, and SOAT1, playing a central role in maintaining systemic lipid homeostasis, while dysregulated lipid metabolism constitutes a critical pathological basis for atherosclerosis development [65].

Currently, the molecular mechanisms underlying the anti-atherosclerotic activity of marine-derived bioactive substances mainly focus on the following aspects: alleviating endothelial injury [66], mitigating macrophage foam cell formation [67], inhibiting smooth muscle cell proliferation [68], and reducing the synthesis of cholesterol [69].

Endothelial dysfunction is the initiating step in atherosclerosis. Marine-derived bioactive substances can alleviate endothelial injury and delay plaque formation by preserving vascular endothelial integrity and inhibiting the expression of adhesion molecules as well as oxidative stress responses [66]. For example, Song et al. [70] utilized Echinochrome A (EchA) to alleviate endothelial injury and established an inflammation-mediated endothelial-to-mesenchymal transition (EndMT) model by co-treating cells with TGF-β2 and IL-1β. Western blot results showed that EchA effectively reversed the upregulation of α-SMA and fibronectin, as well as the downregulation of CD31 and VE-cadherin induced by these cytokines, indicating that EchA inhibits the transition of endothelial cells toward a mesenchymal phenotype. Further investigation of downstream signaling revealed that EchA reduced the phosphorylation levels of NF-κB p-p65, p-IκBα, and p-Smad2/3, suggesting that it exerts its regulatory effects by suppressing the two key signaling pathways, namely the classical NF-κB pathway and the Smad pathway. Since activation of the Rho GTPase pathway is associated with cell migration, EchA also suppressed the expression of RhoA. Moreover, at the level of oxidative stress and mitochondrial function, EchA effectively decreased intracellular ROS production induced by TGF-β2 and IL-1β and upregulated the mRNA expression levels of mitochondrial biogenesis-related genes (including COXII, TUFM, SSBP1, TFB2M, POLG, and TFAM), indicating that it ameliorates mitochondrial dysfunction.

The uptake of oxidized low-density lipoprotein (ox-LDL) by macrophages, leading to foam cell formation, is a key step in the progression of atherosclerosis. Marine-derived bioactive substances can effectively mitigate foam cell formation by downregulating the expression of inflammatory factors and promoting cholesterol efflux [67]. Mirza et al. [71] used THP-1 macrophages as a model and induced foam cell formation with ox-LDL. They found that pretreatment with fucoidan (50 µg/mL) significantly reduced intracellular lipid accumulation and inhibited macrophage foam cell formation. The underlying mechanism involved upregulation of ABCA1 expression at both gene and protein levels, enhanced cholesterol efflux capacity, and increased LXR-α mRNA levels, suggesting that fucoidan may exert its anti-atherosclerotic effects through LXR-α-mediated transcriptional activation of ABCA1. Marasinghe et al. [72] isolated two small peptides, LLRLTDL and GYALPCDCL, from clam shell protein hydrolysate. In ox-LDL-induced RAW264.7 macrophages, these two peptides significantly inhibited foam cell formation. Their molecular mechanism exhibited dual regulatory effects: on one hand, they downregulated the cholesterol influx-related proteins SR-A1 and CD36; on the other hand, they upregulated the cholesterol efflux transporters ABCA1 and ABCG1. In terms of signaling pathways, the two peptides activated the PPAR-γ/LXR-α signaling axis, and the key initiating role of PPAR-γ in this pathway was confirmed through PPAR-γ-specific siRNA transfection and agonist intervention experiments. Furthermore, the two peptides also demonstrated anti-inflammatory activity by inhibiting NF-κB nuclear translocation and reducing the production of pro-inflammatory cytokines. In summary, these two marine-derived bioactive peptides suppress macrophage foam cell formation by activating the PPAR-γ/LXR-α signaling pathway, thereby synergistically regulating cholesterol metabolism and inflammatory responses.

Marine active substances inhibit the excessive proliferation and migration of smooth muscle cells by modulating growth factor signaling pathways and cyclins. Kim et al. [73] found that HMGB1 induces vascular smooth muscle cell (VSMC) migration by activating the AP-1 transcription factor and upregulating osteopontin (OPN) expression. The marine-derived bioactive substance Echinochrome A (Ech A), following pretreatment at 3 μM or 10 μM for 24 h, was shown by chromatin immunoprecipitation (ChIP) assay to significantly inhibit HMGB1-induced binding activity of AP-1 to the OPN promoter, thereby blocking OPN transcription and expression, and ultimately suppressing aberrant VSMC migration in a concentration-dependent manner. Seo et al. [74] used HMGB1-induced rat aortic vascular smooth muscle cells (VSMCs) as a model and found that Echinochrome A (Ech A, 3 μM and 10 μM) inhibited cell proliferation in a concentration-dependent manner. Mechanistic studies showed that Ech A significantly reduced HMGB1-induced increases in the phosphorylation levels of mTOR, p70S6K, and 4E-BP1. Intervention experiments using the PI3K inhibitor LY294002 and the mTOR inhibitor rapamycin further confirmed that Ech A exerts its anti-proliferative effect primarily through inhibition of the PI3K/Akt/mTOR signaling axis.

Marine active substances reduce endogenous cholesterol synthesis by inhibiting HMG-CoA reductase activity and regulating cholesterol metabolism-related transcription factors. Ying et al. [75] used high-fat diet-fed ApoE^−/−^ mice as a model to investigate the anti-atherosclerotic effect of Saringosterol derived from *Sargassum fusiforme* and its underlying molecular mechanism. Saringosterol selectively activates liver X receptor β (LXRβ) in vitro. In contrast, the full LXRα/β agonist T0901317, despite its anti-atherosclerotic activity, induces hepatic lipid accumulation due to concurrent activation of LXRα. This study validated the therapeutic effect of Saringosterol in vivo. The results showed that Saringosterol treatment significantly reduced aortic plaque area and lowered serum total cholesterol levels, without causing hepatic steatosis or elevating transaminase levels, indicating a better safety profile than T0901317. Mechanistically, Saringosterol selectively activates LXRβ and coordinately regulates cholesterol metabolism at multiple organ levels: in the liver, it upregulates CYP7A1, CYP27A1, and ABCG5/G8 to promote cholesterol conversion to bile acids and subsequent biliary excretion; in the small intestine, it downregulates NPC1L1 to reduce cholesterol absorption while upregulating ABCG5/G8 and ABCA1 to enhance cholesterol efflux; in macrophages, it promotes cholesterol efflux and inhibits cholesterol uptake. In summary, this study demonstrates that Saringosterol, as a marine-derived selective LXRβ agonist, effectively alleviates atherosclerosis while avoiding the adverse effect of hepatic lipid accumulation.

Vijay et al. [76] isolated a sulfated polysaccharide, APP-3, from *Arthrospira platensis*. Using Caco-2 cells as a model, they established an in vitro dyslipidemia model induced by oleic acid, and measured triglyceride levels, cholesterol uptake, pancreatic lipase activity, HMGCR expression, and ApoB expression. The results showed that APP-3 at concentrations of 6.25–25 μg/mL concentration-dependently reduced triglyceride levels (by 6–42%), downregulated ApoB expression (from 13.47-fold to 6.98-fold), decreased cholesterol uptake (by 2–44%), and inhibited HMGCR activity (IC_50_ 0.92 mg/mL), indicating that this polysaccharide can effectively regulate lipid metabolism. Liu et al. [77] screened and obtained the polyketide compound enterocin from a marine-derived Streptomyces strain. In vitro studies showed that enterocin directly binds to the ASGR1 protein and promotes its degradation via the ubiquitinproteasome pathway, thereby activating the AMPKα-LXRα signaling axis and upregulating the expression of cholesterol efflux-related transporters such as ABCA1. In vivo experiments demonstrated that enterocin significantly reduces serum triglyceride, total cholesterol, and LDL-C levels, increases HDL-C levels, alleviates hepatic lipid accumulation, and enhances fecal cholesterol excretion. Of note, compared with the positive control drugs atorvastatin (a statin lipid-lowering agent) and GW3965 (an LXR agonist), enterocin exhibited comparable or slightly superior lipid-lowering efficacy in LDLR^−/−^ mice, without affecting normal intestinal lipid absorption, thereby avoiding the potential intestinal side effects commonly associated with statin therapy.

## 5. Clinical Transformation Challenges and Strategies

### 5.1. Complex Components

Active substances found in oceans generally exist as mixtures of diverse chemical components, making it difficult to ascertain which specific active components are genuinely effective. For example, the internal organs of abalones harbor numerous compounds, including polysaccharides, protein peptides, fatty acids, etc. Consequently, separation and identification of effective components present formidable challenges that impose substantial difficulties on quality control and evaluation of efficacy [78]. Moreover, factors such as distinct origins of production, harvest seasons, and extraction methods influence the composition and content of the active substances, leading to unstable product quality and affecting repeatability and reliability of clinical trial results.

Methods for separation, purification, and identification of active components are continuously being improved to identify effective active ingredients. For instance, ultrafiltration is a method used to separate and purify natural products based on membrane separation technology. Its core principle is the use of ultrafiltration membranes with specific pore diameters. Under pressure-driven conditions, separation is achieved based on differences in the rates at which substances of varying molecular weights permeate the membrane. The pore diameters of ultrafiltration membranes typically range from 1 to 100 nm. They can retain large molecules, such as proteins, polysaccharides, and colloids that have molecular weights exceeding the retention value of the membrane pores, whereas small-molecule active components, solvents, and inorganic salts can pass through the membrane, thereby achieving removal of impurities and effective enrichment of components [79]. Ion-exchange chromatography achieves separation of active components by leveraging the varying capacities of the separated components to exchange ions with those of the ion-exchange resin. Cation-exchange resins separate cationic compounds, whereas anion-exchange resins separate anionic compounds [80]. Mass spectrometry (MS) is used to obtain a mass spectrum by ionizing sample molecules and then separating and recording their relative intensities based on the mass-to-charge ratio (m/z). Thus, MS can provide information related to molecular weight, molecular formula, and structural fragments of a compound. In the identification of organic synthesis products, mass spectrometry can rapidly determine whether the molecular weight of a product aligns with the expected value and further determine the structure through analysis of fragment ions [81].

### 5.2. The Mechanism of Action of Ocean-Derived Active Substances Is Unclear

Owing to their intricate composition, it is frequently challenging to elucidate the precise mechanism through which ocean-derived active substances act on the human body. Numerous active substances exert their effects via multiple targets and pathways, and this complex mode of action transcends the scope of traditional single-target drug research. For example, certain active substances may concurrently regulate multiple signaling pathways. However, it remains unclear which pathways are pivotal and how they interact with each other. This makes it difficult to accurately simulate their mechanism of action in preclinical studies and presents challenges in the design and interpretation of clinical trials [82].

Currently, several novel technologies, including proteomics, metabolomics, big data, and artificial intelligence, have emerged. Proteomics and metabolomics can comprehensively analyze the impacts of natural products on proteins and metabolites within the body and disclose their mechanism of action [83,84,85]. Big data and artificial intelligence can integrate a vast amount of research data on natural products, predict active ingredients, screen potential drug targets, assist in the design of clinical trials, and enhance the efficiency of clinical translation [86,87]. Employing these new methods, target sites and signaling pathways of drugs can be detected and their mechanism of action can be clarified.

### 5.3. Poor Pharmacokinetic Properties of Active Substances

Certain active substances encounter issues such as low solubility and suboptimal bioavailability. For example, some lipophilic natural compounds exhibit poor solubility in the gastrointestinal tract, leading to incomplete absorption and the inability to attain an effective blood drug concentration. Moreover, their metabolic processes within the body are highly intricate, and they may be rapidly metabolized or excreted, making it challenging to maintain an effective drug concentration and duration of action, thus influencing their clinical efficacy [88].

In light of the unfavorable pharmacokinetic properties of these active substances, novel dosage forms have been developed. For instance, nano-formulations can augment the solubility and bioavailability of natural products [89]. Formulations such as liposomes and microspheres can achieve targeted drug delivery, extend the duration of drug action in the body, and mitigate toxic side effects [90,91]. Additionally, the development of new oral dosage forms, including immediate-release, sustained-release, and controlled-release preparations, can enhance medication compliance of patients and increase the clinical application value of natural products [92]. Future research may focus on the bioavailability, food matrix interactions, and scalable extraction methods for these marine bioactives.

## 6. Conclusions

In recent years, there has been a continuous increase in reports on marine-derived bioactive substances against atherosclerosis [93]. These active compounds exhibit remarkable chemical diversity and uniqueness. For example, fucoxanthin, a carotenoid derived from marine sources, possesses an extended conjugated polyene chain and terminal polar groups, enabling it to integrate into lipid membranes and quench reactive oxygen species in situ [94]. Another example is 24(S)-saringosterol from *Sargassum fusiforme*, a sterol derivative with an S-configuration at C24. Chen et al. [95] separated the epimers by semi-preparative HPLC and, using luciferase reporter gene assays, found that it activated LXRβ with a transcriptional activation fold of 3.50 ± 0.17 and effectively induced the expression of cholesterol reverse transport genes such as ABCA1, ABCG1 and ApoE, confirming its selective LXRβ-agonistic anti-atherosclerotic activity. In terms of structural characterization, researchers have employed modern spectroscopic techniques including NMR, mass spectrometry, X-ray diffraction and electronic circular dichroism (ECD) to elucidate the structures and absolute configurations of a large number of marine compounds. Notably, significant progress has been made in the complete structural characterization of anti-atherosclerotic substances [96]. For instance, in 2016, Verdan H. et al. [97] isolated a novel tetracyclic sesquiterpene, 19-methoxy-9,15-ene-puupehenol, and its analogue from the Australian sponge *Hyrtios digitatus*. Using ^1^H and ^13^C NMR combined with ECD data, they successfully determined their unique structures and absolute configurations. These compounds exhibited anti-atherosclerotic activity in an SR-B1 overexpressing cell model with IC_50_ values of 1.78 μM and 3.05 μM, respectively. Regarding molecular targets, the elucidated targets of marine compounds include multiple key nodes involved in lipid metabolism regulation (e.g., LXRα, SR-B1, PCSK9), inflammatory pathways (e.g., NF-κB, PI3K/Akt), oxidative stress (e.g., Nrf2, HO-1) and endothelial function (e.g., Akt/eNOS) [98,99,100,101,102]. To date, many marine substances have demonstrated anti-atherosclerotic efficacy in in vivo experiments. For example, Eguchi K. et al. administered manzamine A orally to ApoE-knockout mice for 80 consecutive days and observed significant reductions in serum total cholesterol, free cholesterol, LDL cholesterol and triglyceride levels, as well as a marked decrease in atherosclerotic plaque area in the aortic sinus, confirming that manzamine A exerts anti-atherosclerotic effects in vivo by inhibiting ACAT activity [103]. However, the clinical translation of this field still faces several challenges. For instance, marine polysaccharides such as fucoidan suffer from structural variability and low oral bioavailability [104]; the marine sulfated polysaccharide propylene glycol alginate sodium sulfate (PSS) has poor oral absorption due to its high molecular weight and degradation in the gastric acid environment [105]. Future efforts should focus on improving pharmacokinetic properties through chemical structure optimization and the development of novel delivery systems such as enteric-coated nanoparticles, advancing multi-target combination therapies, and simultaneously establishing sustainable chemical synthesis or synthetic biology production methods, in order to propel marine bioactive substances toward the development of new anti-atherosclerotic drugs.

## Data Availability

The data analyzed in this review are from previously published studies, which are listed in the references. The original datasets can be accessed via the respective publications or the public repositories mentioned therein.

## Abbreviations

The following abbreviations are used in this manuscript:

TC	Total Cholesterol
TG	Triglyceride
HDL-C	High-Density Lipoprotein Cholesterol
LDL-C	Low-Density Lipoprotein Cholesterol
FC	Free Cholesterol
CE	Cholesteryl Ester
IL-6	Interleukin-6
IL-1β	Interleukin-1β
IL-10	Interleukin-10
TNF-α	Tumor Necrosis Factor-α
SOD	Superoxide Dismutase
MDA	Malondialdehyde
ROS	Reactive Oxygen Species
GSH	Glutathione
NO	Nitric Oxide
ICAM-1	Intercellular Cell Adhesion Molecule-1
VCAM-1	Vascular Cell Adhesion Molecule-1
MCP-1	Monocyte Chemoattractant Protein-1
SR-A1	Scavenger Receptor A1
ABCA1	ATP Binding Cassette Transporter A1
PCSK9	Proprotein Convertase Subtilisin/Kexin Type 9
ABCG5	ATP Binding Cassette Transporter G5
LDLR	Low-Density Lipoprotein Receptor
Caspase-3	Cysteinyl aspartate specific proteinase-3
